# A Review on Gas Indicators and Sensors for Smart Food Packaging

**DOI:** 10.3390/foods13193047

**Published:** 2024-09-25

**Authors:** Wonyoung Heo, Seokwon Lim

**Affiliations:** Department of Food Science & Biotechnology, Gachon University, 1342 Seongnam-daero, Sujeong-gu, Seongnam-si 13120, Republic of Korea; hwy981@gmail.com

**Keywords:** smart packaging, indicators, sensors, oxygen, carbon dioxide, ammonia

## Abstract

Real-time monitoring of changes in packaged food is crucial to ensure safety and alleviate environmental issues. Accordingly, the development of indicators and sensors for smart packaging has long been anticipated, especially for gases related to food deterioration and microbial growth. However, the characteristics of indicators and sensors used in food packaging cannot be adjusted according to the specific food type, making it essential to select and apply suitable indicators and sensors for each type of food. In this review, the principles and characteristics of gas indicators and sensors for oxygen, carbon dioxide, and ammonia that are commercialized or in the development phase were summarized, and their application status and prospects were assessed. Indicators and sensors for smart packaging are applied in forms such as films, labels, sachets, and devices. Their detection methods include redox reactions, analyte binding, enzyme reactions, pH changes, electron transfer, conformational changes, and electrode reactions. In this work, 9 types of indicators and sensors for oxygen, carbon dioxide, and ammonia were evaluated based on their detection and indication methods, materials, sensitivity, detection range, limit of detection, and advantages and disadvantages in food applications. We anticipate our review will propose criteria for selecting the optimal indicators and sensors for specific foods. Furthermore, this review examines the current application status and future prospects of these indicators and sensors.

## 1. Introduction

Food packaging is essential for preventing spoilage and damage by protecting food from external factors, thereby delaying deterioration, extending shelf life, and ensuring quality and safety. Additionally, food packaging serves as a platform for providing consumers with detailed product information, including nutritional content, health benefits, usage and storage instructions, manufacturing location, production date, expiry date or best-before date, and other relevant details. This enables consumers to make informed decisions about the food products they purchase [1,2,3,4,5,6,7,8,9,10,11,12,13,14].

In recent years, however, growing consumer concern over food quality and safety has led to significant changes in food distribution and consumption. This shift has driven advancements in packaging technologies aimed at providing more detailed information about food quality [3,4,9,10,12,13,14,15,16,17,18,19,20]. Therefore, smart packaging utilizing active and intelligent packaging technologies has been attracting attention. Active packaging is a packaging technology that increases the shelf life of food by interacting with food or the internal environment of the package [1,2,5,7,8,9,10,11,21,22]. Intelligent packaging is a packaging technology that monitors changes in food or the internal environment of the package and provides information about it [2,3,4,7,9,10,11,14,18,19,21,22,23,24,25,26].

In the past, active packaging, designed to ensure food quality and safety and extend shelf life, received more attention than intelligent packaging. However, in recent years, advancements in information technology and the growing need to manage the food supply chain have led to a remarkable increase in interest in intelligent packaging [3,7,9,10,22,27]. Additionally, the rising interest in intelligent packaging can be attributed to the growing number of research projects, development initiatives, and patents related to intelligent technology. Its potential to verify the effectiveness of packaging technologies, such as active packaging, also contributes to this increased interest [8,27].

In line with these trends, this review aims to explore the significance and potential applications of intelligent packaging technologies, with focusing on gas indicators and sensors. It is essential to ensure that these indicators and sensors are compatible with the food being monitored, as not all of them can be applied to every type of food [3,28]. Therefore, determining the appropriate indicator or sensor for each specific product is crucial [28]. To achieve this, we investigated currently commercialized and developing indicators and sensors, thoroughly analyzing the principles and characteristics of each technology. The articles and patents reviewed were selected using combinations of the following keywords: ‘oxygen’, ‘carbon dioxide’, ‘ammonia’, and either ‘indicator for food packaging’ or ‘sensor for food packaging’. These gases were chosen because they are major markers of food quality [11,18,29]. The literature search was conducted using Google Scholar for peer-reviewed articles published between 2004 and 2022. For patent analysis, we used the KEYWERT database to search patents published or registered in South Korea, Japan, the United States, and Europe up to 16 June 2022.

To provide a comprehensive overview, the study categorizes these indicators and sensors based on their detection method, indication method, sensitivity, and detection range or limit of detection (LOD). This classification highlights the unique features of each technology and its potential to ensure food quality and safety. Based on this analysis, we have proposed information to help establish criteria for selecting the optimal indicators and sensors for specific food applications and evaluate the current applications and future prospects of these technologies.

## 2. Intelligent Packaging Systems

Intelligent packaging is a system designed to maintain the quality and value of food products by monitoring their internal environment and status. It tracks product movement through the supply chain and records relevant information during transportation and storage. This system provides crucial data on the quality, safety, and history of the food throughout its entire journey [2,3,4,7,8,9,10,18,19,21,25,26,30].

The intelligent packaging system is capable of performing six key functions: monitoring, detecting, sensing, recording, tracking, and communicating [1,2,3,7,8]. To perform these functions, intelligent packaging utilizes three key systems: indicators, sensors, and data carriers. Indicators and sensors detect changes in the product or its environment through specific mechanisms, providing this information via visual changes. Data carriers, such as barcodes and radiofrequency identification tags (RFID), store information about the production, processing, and distribution stages of the product, allowing users to access this information by scanning it [2,3,7,8].

## 3. Gas Indicators and Sensors

**Figure 1:**
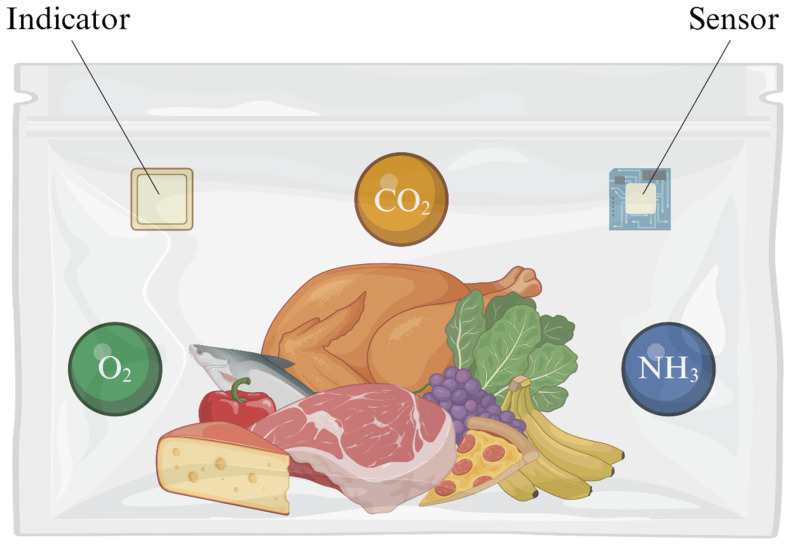
Illustration of food packaging with indicators and sensors attached inside the packaging to detect oxygen, carbon dioxide, or ammonia. Indicators show gas presence through color changes, while sensors provide quantitative data by converting this information into electrical signals. This system monitors microbial activity and environmental changes in real time [1,2,3].

Indicators and sensors detect specific analytes and transmit information about their presence. These devices are used in intelligent packaging to provide data on microbial activity within the package and changes in the product or its environment (Figure 1). They function based on characteristics such as the presence or absence of a target chemical or biological substance, reactions between substances, variations in substance concentration, and potential biological processes and reactions [1,2,3,11,14]. As previously mentioned, gas indicators and sensors can detect gases such as oxygen, carbon dioxide, and ammonia, which are key markers of food quality (Figure 1).

An indicator is a device that provides information about the presence of an analyte, along with qualitative and quantitative data, by converting it into a visible form, such as a color change [14,20,23]. Indicators used in smart packaging can be classified into external and internal indicators based on their attachment form. External indicators function by being attached to the exterior of the packaging, while internal indicators operate by being attached inside the packaging [3,23,31].

Unlike an indicator, a sensor consists of a receptor and a transducer. The receptor detects the presence, activity, composition, or concentration of specific analytes. The physical or chemical information measured by the receptor is then converted into a form of energy that can be measured by the transducer. Subsequently, the transducer converts this signal into an analytical signal, which is then displayed [2,3,8,14,28].

Devices that provide information about food quality through the analysis of the internal atmosphere in food packaging can be referred to as gas indicators and sensors. These devices detect gases generated from enzymatic reactions, chemical reactions, and microbial metabolism within the food, as well as gases entering the package from the external environment, and provide this information to the user [2,4,7,8,22,27,28,32,33].

### 3.1. Oxygen Indicator and Sensor

**Figure 2:**
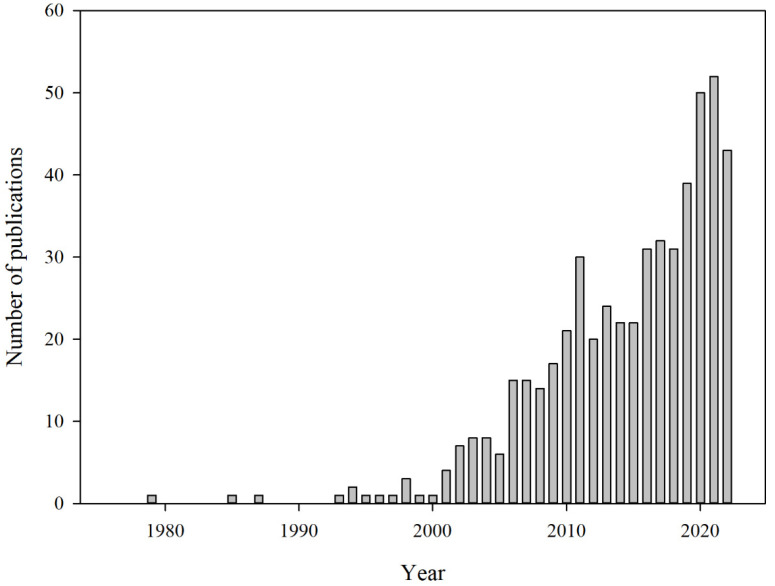
The annual number of publications related to oxygen indicators and sensors for food packaging has been indexed in PubMed up to 2022 [47]. The number of publications shows a significant increase, particularly after 2000, reflecting growing research interest in this field.

When packaging designed to block air, such as Modified Atmosphere Packaging (MAP), commonly used for ready-to-eat foods that require extended shelf life, is compromised, oxygen from the outside enters, leading to an increase in the oxygen concentration inside the package [11,18,25,26,29,34,35,36]. This influx of oxygen allows microorganisms, enzymes, and other organisms within the package or food to accelerate spoilage and trigger enzymatic browning reactions in fruits and vegetables through metabolic activity. Additionally, the presence of oxygen contributes to the oxidation of vitamin C and lipids [13,18,25,29]. Therefore, oxygen is a critical factor in food deterioration, and extensive research is being conducted on the development of oxygen indicators and sensors to detect and monitor it (Table 1, Figure 2) [18,19,29].

**Table 1:** Investigated oxygen indicators and sensors.

Indicator/ Sensor	Detection Method	Indication Method	Sensitivity		Detection Range or LOD		Reference
Colorimetric redox dye-based indicator	Redox reaction	Color change	N/A		0.5% (Ageless eye™)		[10,26,29,37]
Oxygen-binding complexes-based colorimetric indicator	Oxygen binding	Color change	Deoxyhemoglobin	20–100 Torr	N/A		[29,38]
			Myoglobin	2–8 ppm			
Pressure-activated compartmented oxygen indicator	Enzyme reaction	Color change	N/A		N/A		
Photoluminescence-based oxygen indicator	Molecular collision	Quenching of luminescence intensity	Silicone rubber (RTV 118)	30 Torr	In water	15 µmol/dm^3^	[29,39]
			Poly(vinyl chloride)	132 Torr	In gas	0.03%	
			Polystyrene	495 Torr			
Light-activated colorimetric redox dye-based oxygen indicator	Redox reaction	Color change	N/A		0.5%		[25,26,29,34,35,36,40,41,42,43,44,45]
Self-powered flexible oxygen gas sensor	Redox reaction	Electrical signal	18 mV/% O_2_		0–21%		
Organic/inorganic hybrid compound-based oxygen indicator	Redox reaction	Color change	Under 0.1% O_2_	Pink	0.1%		[46]
			Over 0.5% O_2_	Blue			
Self-powered oxygen/temperature indicator-sensor using film-type battery	Redox reaction	Color change	N/A		N/A		
Activation controlled oxygen indicator	Redox reaction	Color change	N/A		N/A		

N/A: Not Available.

#### 3.1.1. Colorimetric Redox Dye-Based Indicator

**Figure 3:**
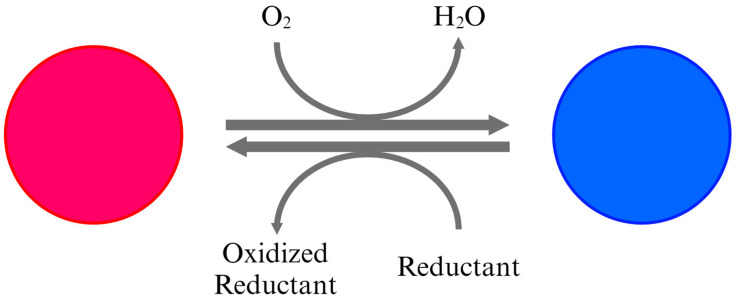
The detection and indication mechanism of colorimetric redox dye-based indicators operates based on a redox reaction that involves a color change in the dye. In the absence of oxygen, the reducing agent in the indicator reduces the redox dye, typically methylene blue, causing it to change color. When oxygen is present, it oxidizes the dye, reverting it to its original color [25,26,29,37].

As the most representative form of oxygen indicators, most commercial oxygen indicators are based on redox dyes. Typically, these indicators use a detection mechanism involving a redox dye like methylene blue, a reducing agent, and an alkaline solution. When oxygen is present, the redox dye changes color, while in the absence of oxygen, the reducing agent causes the indicator to remain colorless in the alkaline solution (Figure 3) [25,26,29,37]. These indicators have the advantage of easily detecting the presence of oxygen through a color change in the dye without the need for special equipment or expertise, and they are inexpensive to produce. However, they have three major problems. First, the indicator can be prematurely activated by reacting with oxygen in the air before being placed inside the package. For accurate detection, the indicator should activate only after it has been inserted into the package and the package is sealed, accounting for the environmental changes inside. However, common redox dye-based indicators are less accurate because they tend to activate before being inserted into the package. Another problem is the reversible response of the indicator. When the packaging is damaged, oxygen from the outside air infiltrates into the package, causing a color change in the indicator. However, the indicator can revert to its original color when microorganisms or enzymes within the package consume oxygen for metabolism, decreasing the internal oxygen concentration and complicating accurate oxygen analysis. The last problem concerns the safety of materials used in the indicator. Since the indicator is attached inside the packaging, it can come into direct contact with the food. The materials composing the indicator may dissolve due to the moisture in the food, potentially contaminating the food product. If these materials are harmful to human health, they could pose a risk to consumer safety. Studies are being conducted to address these problems. One primary approach is to coat the indicator with a polymer. This method can prevent the indicator materials from eluting due to moisture. One investigated indicator, Ageless Eye™, a commercially available product, has a detection limit of 0.5% oxygen [10,26,29,37].

#### 3.1.2. Oxygen-Binding Complexes-Based Colorimetric Indicator

To address the safety concerns associated with traditional indicators, new indicators utilizing natural compounds are being developed, avoiding the use of synthetic dyes commonly found in conventional indicators. Similar to standard colorimetric indicators, oxygen-binding complex-based indicators detect the presence of oxygen through a color change. This mechanism relies on the color change that occurs when oxygen binds to the oxygen-binding complex, such as in hemoglobin or myoglobin. These complexes change color upon oxygen binding. Indicators made with metmyoglobin function by reducing metmyoglobin to deoxymyoglobin using a reducing agent. Deoxymyoglobin then binds with oxygen in the presence of oxygen, resulting in a color change. While this allows for easy detection of oxygen through color change, such indicators suffer from poor storability, lasting only about two days at room temperature. They also tend to react with oxygen in the air before application inside packaging, and their reactions are reversible. To mitigate these issues, one approach is to encapsulate the oxygen-binding complex within a sol-gel glass matrix. However, this method reduces the indicator’s color-changing ability and remains problematic due to sensitivity to pH and humidity changes. Additionally, the indicator’s reaction remains reversible. When deoxyhemoglobin is used, the indicator’s sensitivity ranges from 20 to 100 Torr of oxygen, whereas myoglobin offers a sensitivity of 2 to 8 ppm. In terms of storability, myoglobin has been found to be more stable than hemoglobin [29,38].

#### 3.1.3. Pressure-Activated Compartmented Oxygen Indicator

**Figure 4:**
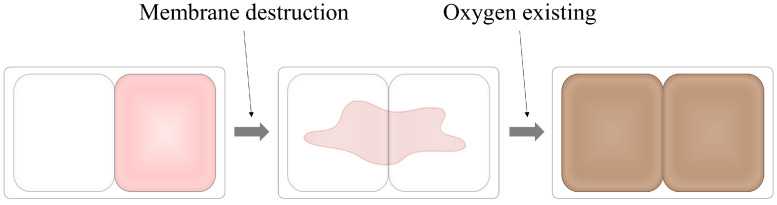
The pressure-activated compartmented oxygen indicator functions by destroying the membrane that separates the components. This allows the oxidase enzyme to react with the substrate in the presence of existing oxygen, causing a color change to indicate oxygen presence [18].

Conventional colorimetric indicators posed safety concerns as the indicator materials could leach and infiltrate into food due to moisture. Additionally, these indicators are often activated prematurely due to exposure to oxygen in the air before being applied to packaging. To address these issues, a pressure-activated compartmented oxygen indicator was developed. This new indicator detects the presence of oxygen through color changes in its constituents. The detection mechanism relies on the conversion of a substrate (guaiacol) to a product of a different color by an oxidase (laccase). The oxidase converts the substrate, which has been reduced by a reducing agent (cysteine), into the product using oxygen generated inside the package (Figure 4). This indicator offers the advantage of being safe since it utilizes natural compounds instead of synthetic or chemical substances. Furthermore, the materials constituting the indicator are separated by a physical barrier, allowing activation through the application of physical force. This means the indicator can be activated at the desired time, providing greater control and safety [18].

#### 3.1.4. Photoluminescence-Based Oxygen Indicator

**Figure 5:**
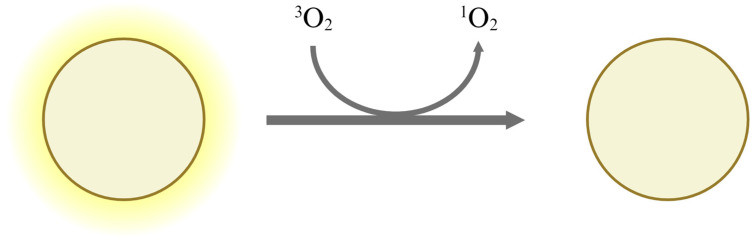
The detection and indication mechanism of photoluminescence-based oxygen indicator. When the UV-energized dye collides with ground-state oxygen (triplet oxygen), it transfers energy to the oxygen, causing it to reach an excited state (singlet oxygen). The dye then returns to its ground state, resulting in reduced light intensity [29,39].

Existing oxygen indicators primarily use colorimetric dyes that change color in the presence of oxygen. However, fluorescent dyes, which indicate the presence of oxygen through changes in light intensity rather than color, are also used. Photoluminescence-based oxygen indicators are created by encapsulating fluorescent dyes in silicon or organic polymers, such as vinyl chloride, which are gas-permeable but ion-impermeable. The detection mechanism involves collisional quenching, which occurs when a fluorescent dye in an excited state collides with oxygen molecules [29,39,48]. When short-wavelength light, such as UV rays, irradiates the fluorescent dye, energy is supplied to the dye, putting it into an excited state and causing it to emit light. If the excited fluorescent dye collides with an oxygen molecule in its ground state (triplet oxygen), the oxygen molecule absorbs energy from the dye and transitions to an excited state (singlet oxygen). Consequently, the fluorescent dye returns to its ground state, resulting in a reduction in light intensity (Figure 5). Photoluminescence-based oxygen indicators remain inactive until UV irradiation, allowing control over the activation time. This process is irreversible. Because the components are encapsulated within polymers, they are protected from leakage due to moisture. When using [Ru(dpp)_3_](ClO_4_)^2^, sensitivity varies depending on the encapsulation material. Silicon rubber (RTV118) has a sensitivity of 30 Torr, poly(vinyl chloride) has 132 Torr, and polystyrene has 495 Torr. Among the commercially available products, O2xyDot™ has a detection range of up to 15 µmol/dm^3^ in water and 0.03% in gas [29,39].

#### 3.1.5. Light-Activated Colorimetric Redox Dye-Based Oxygen Indicator

**Figure 6:**
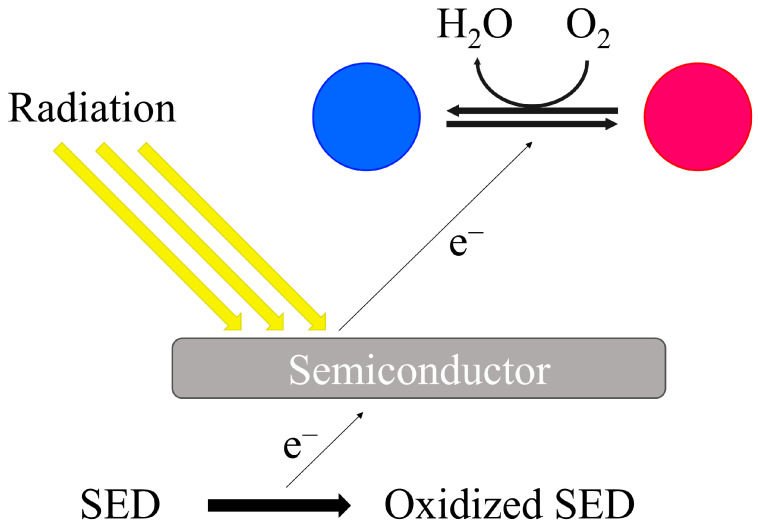
The detection and indication mechanism of light-activated colorimetric redox dye-based oxygen indicator. The semiconductor absorbs high-energy light, generating electron holes and electrons. Electron holes oxidize the electron donor, transferring electrons to the redox dye and causing a color change. In the presence of oxygen, the dye reverts to its original color, indicating the presence of oxygen [25,26,29,34,35,36,40,41,42,43,44,45].

One of the extensively researched types of oxygen indicators is the light-activated colorimetric redox dye-based oxygen indicator. This indicator addresses issues associated with traditional redox dye-based indicators. It is created by dispersing a semiconductor, electron donor, redox dye, and coating polymer in an organic solvent, and then applying this mixture to a film. Like other colorimetric indicators based on redox dye, the presence of oxygen is indicated by a color change. The detection mechanism also involves color changes caused by the oxidation of the redox dye, but unlike conventional redox dye-based colorimetric indicators, it involves electron transfer between the semiconductor, electron donor, and redox dye. When the semiconductor absorbs high-energy light, it becomes activated, generating electron holes and electrons. The electron holes receive electrons by irreversibly oxidizing the electron donor; then, electrons are transferred to the redox dye to reduce it; therefore, the color of the dye changes. In the presence of oxygen, the color of the reduced dye reverts to its original state (Figure 6). This means that the indicator is only activated when exposed to high-energy light, preventing premature activation before packaging. Additionally, the safety of the components is ensured because they are coated with a polymer, preventing elution by moisture. Furthermore, the accuracy of the indicator is high due to the irreversibility of the reaction [25,26,29,35,36,40,41,42,43,44,45].

In the case of an indicator made using methylene blue, glycerol, titanium dioxide, and EVOH, it has a LOD of 0.5% oxygen [25].

#### 3.1.6. Self-Powered Flexible Oxygen Gas Sensor

**Figure 7:**
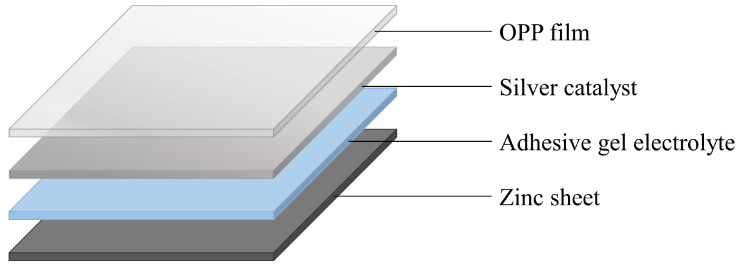
Self-powered flexible oxygen gas sensor components. The components include a metal cathode (zinc sheet), air anode (silver catalyst), and electrolyte (adhesive gel electrolyte), with the OPP film as the top layer [19].

Some indicators and sensors developed for food packaging applications face limitations due to improper size, toxicity of components, and high manufacturing costs. To overcome these challenges, a self-powered, flexible oxygen gas sensor using a metal-air battery was developed. This sensor detects oxygen and converts it into an electrical signal to indicate its presence. The detection mechanism involves electric current generation due to the oxidation of the metal-air battery’s electrodes. The metal-air battery comprises a metal cathode, air anode, and electrolyte (Figure 7). When oxygen interacts with the anode’s catalyst, the catalyst facilitates the reduction of oxygen to produce hydroxide ions. These hydroxide ions then transfer to the cathode through the electrolyte, where they oxidize the metal cathode, generating electrons. The electrons move to the anode, causing an electric current to flow and thus generating electric power. The sensor is light, compact enough to be applied inside packaging, does not require an external power supply, is non-toxic, and has low manufacturing costs. The sensitivity of the sensor was measured at 18 mV/% O₂, with a detection range from 0 to 21% O_2_ [19].

#### 3.1.7. Organic/Inorganic Hybrid Compound-Based Oxygen Indicator

**Figure 8:**
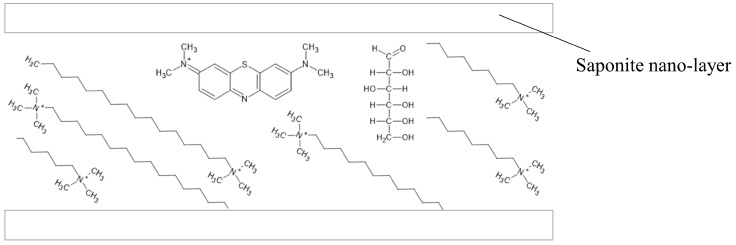
Schematic diagram of organic/inorganic hybrid compound-based oxygen indicator. The diagram shows the organic/inorganic hybrid compound-based oxygen indicator with components such as methylene blue and a cationic surfactant intercalated between saponite nano-layers [46].

Colorimetric redox dye-based indicators often lose their color or ability to change color during storage due to factors like photo-fading. To address this issue, an organic/inorganic hybrid compound-based oxygen indicator was developed. This indicator is created by intercalating components such as methylene blue and a cationic surfactant between the layers of nano-layered compounds like clay minerals (Figure 8). Similar to traditional colorimetric redox-dye indicators, it indicates the presence of oxygen through a color change, utilizing the same detection mechanism as described in Section 3.1.1. The storage stability of this indicator against light and heat is improved, and oxygen diffusion is restricted by the nano-layers, enabling better color preservation of the redox dye. Additionally, the cationic methylene blue is combined with negatively charged clay minerals to prevent D-D type photo-fading. The indicator turns pink when the oxygen level is below 0.1%, and it becomes blue when the oxygen level exceeds 0.5%. The LOD is 0.1% O_2_ [46].

#### 3.1.8. Self-Powered Oxygen/Temperature Indicator-Sensor Using Film-Type Battery

A sensor converts information about the presence of an analyte into an electrical signal through interaction with the analyte. Consequently, additional equipment, such as a display, is required, and power is necessary for operation. However, integrating such equipment into food packaging is challenging. To address this issue, a sensor incorporating a film-type reduction electrode and an oxidation electrode coated with redox dye was developed. This sensor indicates the presence of oxygen through a color change and a voltage difference caused by electron transfer; additionally, it can measure temperature. The detection mechanism relies on the principle that electrons transfer from the oxidation electrode to the reduction electrode as the dye is oxidized by oxygen, which then reduces the oxidized dye. This process results in a difference between the oxidation rate and the reduction rate, allowing oxygen or temperature to be detected through either a color change or a voltage difference. It was confirmed that the sensor’s voltage increased with rising temperature. The sensor is lightweight, with a thin film and a simple configuration, making it easy to manufacture and apply. Furthermore, it does not require an additional power supply [49].

#### 3.1.9. Activation Controlled Oxygen Indicator

Similar to the pressure-activated compartmented oxygen indicator described in Section 3.1.3, this indicator is designed by separating its components [18,50]. This configuration allows for the control of activation timing, preventing unintended activation by oxygen in the air. Additionally, by separating the reducing sugar from the basic substance prevents the browning of the reducing sugar caused by the basic substance, thus addressing the instability issues of some dyes [50].

### 3.2. Carbon Dioxide Indicator and Sensor

**Figure 9:**
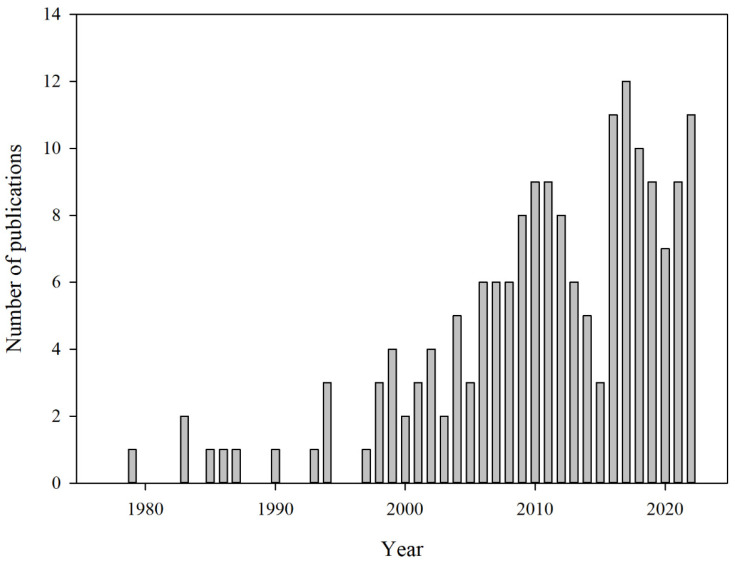
The annual number of publications related to carbon dioxide indicators and sensors for food packaging has been indexed in PubMed up to 2022 [59]. The data show a steady increase in the number of publications, particularly after 2000, indicating growing research interest in this area.

Metabolic processes that occur in food, caused by the presence of intrinsic enzymes or the growth of microorganisms, may generate carbon dioxide or other metabolites. [11,13,15,16,23]. Food deterioration due to microbial metabolic activity can be divided into desirable and undesirable processes. While fermentation can be beneficial in some cases, such as in the production of certain fermented foods, not all fermentation processes are desirable during food storage [11,15,16,23,51,52]. However, these processes share the common feature of producing carbon dioxide, which can be measured to assess the freshness and quality of the food. For this reason, carbon dioxide is regarded as an important marker of food spoilage, and significant research is being conducted on carbon dioxide indicators and sensors that can help consumers easily monitor food freshness (Table 2, Figure 9) [15,16,23,53].

**Table 2:** Investigated carbon dioxide indicators and sensors.

Indicator/ Sensor	Detection Method	Indication Method	Sensitivity	Detection Range or LOD	Reference
Paper-based fluorescent carbon dioxide indicator	Protonation	Color change	N/A	5.7–410 ppm	[11,54]
Chitosan-based irreversible colorimetric carbon dioxide indicator	pH change	Transparency change Color change	N/A	N/A	[16,52]
BTB^−^/TBA^+^ ion-paired dye-based carbon dioxide indicator	pH change	Color change	N/A	N/A	[23]
Whey Protein Isolate (WPI)-based carbon dioxide indicator	pH change	Transparency change	N/A	N/A	
Conducting polymer carbon dioxide indicator	pH change	Electrical signal	N/A	Up to 2455 ppm	
Citric acid-treated chitosan-based carbon dioxide indicator	pH change	Transparency change	N/A	N/A	
Sodium caseinate, pectin-based carbon dioxide indicator	pH change	Transparency change	N/A	N/A	[55,56]
Cresol red anion/tetraalkylammonium cation ion pair-based carbon dioxide indicator	Ionic reaction pH change	Color change	N/A	N/A	[11,57]
Film type acidity indicator	pH change	Color change	N/A	N/A	[11,58]

#### 3.2.1. Paper-Based Fluorescent Carbon Dioxide Indicator

Paper-based indicators have been widely studied due to their versatility and low fabrication costs. Fluorescent-based carbon dioxide indicators have a simple structure and are convenient to use, but they are difficult to apply due to detection limitations and chemical instability. To address these issues, a paper-based fluorescent carbon dioxide indicator has been developed. The indicator uses bis(4-pyridyl)dineopentoxyl-p-phenylenedivinylene (Np-P4VB) as a fluorescent dye, which indicates the presence of carbon dioxide by changing color through fluorescence wavelength conversion. The detection mechanism is fluorescence wavelength conversion due to the protonation of Np-P4VB. Np-P4VB is a synthetic color development group mainly used for its stability, fluorescence, and color-changing characteristics. When carbon dioxide reacts with water, it forms carbonic acid [11,54,60]. The carbonic acid protonates the nitrogen atoms in the two terminal pyrimidine rings of Np-P4VB through interaction with its pyridine ring. As a result, the fluorescence of Np-P4VB switches from turquoise to orange. The indicator has the advantage of having a low LOD and reacting quickly. The detection range of the indicator is from 5.7 to 410 ppm [54].

#### 3.2.2. Chitosan-Based Irreversible Colorimetric Carbon Dioxide Indicator

**Figure 10:**
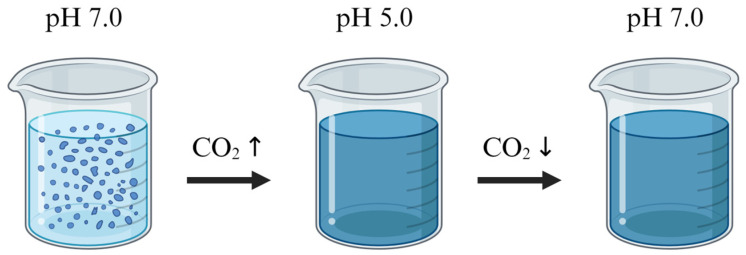
The detection and indication mechanism of chitosan-based irreversible colorimetric carbon dioxide indicator. At pH 7.0, the chitosan is aggregated, and the indicator appears opaque. As carbon dioxide levels increase, the pH decreases to 5.0, causing the chitosan to dissolve and release the encapsulated blue pigment, turning the indicator blue. Even if carbon dioxide levels decrease and the pH increases again, the indicator remains blue, indicating its irreversibility [16].

Makgeolli is a traditional liquor made from fermented rice with nuruk as a starter. Carbon dioxide generated during the fermentation and storage of makgeolli can be used as a marker of its quality. Therefore, a chitosan-based irreversible colorimetric carbon dioxide indicator was developed to easily provide consumers with information on the degree of fermentation of makgeolli. This indicator shows the presence of carbon dioxide through changes in color and transparency. The detection mechanism is based on the solubility of chitosan according to pH changes. The indicator is made by encapsulating an edible blue pigment with chitosan, which is insoluble under neutral conditions (pH 7), making the indicator opaque at pH 7 or higher [16,52]. When carbon dioxide is produced, the pH decreases, causing the chitosan to dissolve in water, releasing the blue pigment encapsulated within. As a result, the indicator becomes transparent and blue (Figure 10). Using only chitosan to indicate the presence of carbon dioxide by changing transparency can be problematic because if the pH increases again, the chitosan reaggregates, making it difficult to accurately determine whether carbon dioxide was generated. However, the indicator developed in this study overcomes this issue. The blue pigment does not re-encapsulate even if the chitosan reaggregates, making the process irreversible (Figure 10). This irreversibility provides high accuracy in detecting carbon dioxide. Although the primary purpose of this indicator is to measure the quality of makgeolli, it is also applicable to other food packaging. It is expected to be useful for monitoring the freshness and spoilage of food during storage, transport, and distribution [16].

#### 3.2.3. BTB^−^/TBA^+^ Ion-Paired Dye-Based Carbon Dioxide Indicator

Due to the advantage of allowing analysis with the naked eye without the need for special equipment, optical detection methods are widely used in gas indicators. Typically, these methods employ dyes; however, several issues arise with their use. If the dye is hydrophilic, it can infiltrate food due to moisture within the packaging. Additionally, these dyes do not mix well with the hydrophobic polymers commonly used in packaging materials. To address these issues, a BTB^−^/TBA^+^ ion-paired dye-based carbon dioxide indicator has been developed. This indicator changes color based on the concentration of carbon dioxide without the influence of moisture. The detection mechanism involves a color change in BTB according to pH levels: BTB appears blue at pH 7.6 (basic condition) and yellow at pH 5.8 (acidic condition). The indicator contains an ion-paired dye and branched polyethylene amine. Polyethylene amine enhances the sensitivity of the indicator. It contains primary amines that react with carbon dioxide to form carbamic acid, which decreases the pH and consequently changes the indicator’s color. The advantages of this indicator include the hydrophobic nature of the ion-paired dye, which reduces the risk of elution by moisture in the food, and the compatibility of the hydrophobic components of the packaging materials and the indicator, ensuring they mix well without compromising the food package. Additionally, while traditional colorimetric carbon dioxide indicators rely on the reaction of carbon dioxide with water to form carbonic acid (lowering pH and changing the indicator’s color), the BTB^−^/TBA^+^ ion-paired dye-based carbon dioxide indicator can rapidly detect carbon dioxide without the need for moisture [23].

#### 3.2.4. Whey Protein Isolate (WPI)-Based Carbon Dioxide Indicator

**Figure 11:**
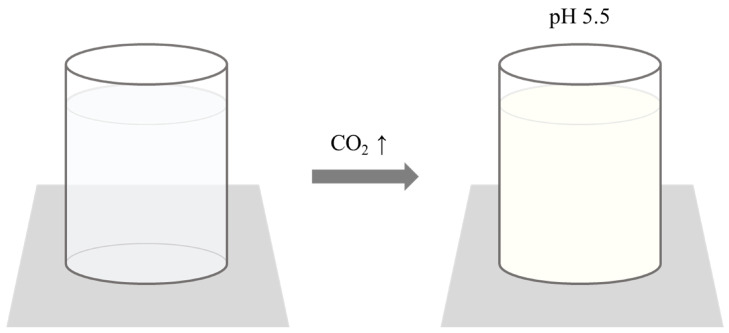
The detection and indication mechanism of WPI-based carbon dioxide indicator. The indicator becomes opaque as carbon dioxide concentration increases, lowering the pH to 5.5 and reducing WPI solubility [15].

Among the indicators mentioned, a whey protein isolate (WPI)-based carbon dioxide indicator has been developed, similar to the chitosan-based irreversible colorimetric carbon dioxide indicator mentioned in Section 3.2.2, which utilizes pH-dependent solubility [15,16]. This WPI-based indicator reveals the presence of carbon dioxide through changes in transparency. The detection mechanism relies on the solubility of WPI, which varies with pH changes. WPI consists of proteins such as β-lactoglobulin and α-lactalbumin found in whey produced during the cheese-making process. Under neutral conditions, WPI is transparent; however, its solubility decreases near its isoelectric point of pH 5.5, causing it to become opaque. Thus, the principle of this indicator is based on the reaction of carbon dioxide with the water-soluble solvent of the indicator, leading to a pH decrease and a corresponding change in transparency (Figure 11). Even if the pH increases again, the indicator exhibits hysteresis, making its transparency change irreversible. This feature provides the advantage of permanently indicating the presence of carbon dioxide. Additionally, this indicator allows for the detection of carbon dioxide without requiring special equipment or expertise [15].

#### 3.2.5. Conducting Polymer Carbon Dioxide Indicator

**Figure 12:**
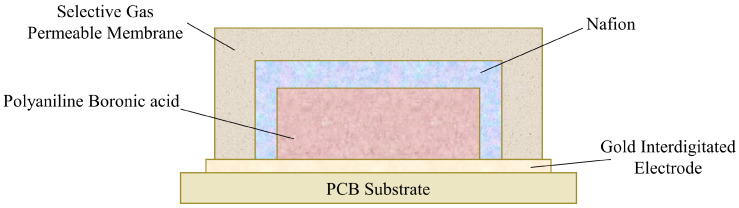
Schematic diagram of the conducting polymer carbon dioxide indicator. The sensor consists of a PCB substrate with a gold interdigitated electrode. Above the electrode is a layer of PABA, surrounded by Nafion. The outermost layer is a selective gas-permeable membrane [61].

Grain can be contaminated by carbon dioxide, moisture, and heat generated during the metabolism of grains, fungi, insects, and mites during storage, resulting in unpleasant odors. A sensor was developed to monitor the change in grain quality by detecting the presence of carbon dioxide. This sensor uses a conductive polymer and indicates the presence of carbon dioxide through a converted electrical signal when the inductor detects it. The detection mechanism relies on the increased conductivity due to the protonation of polyaniline boronic acid (PABA). PABA is sensitive to pKa changes, which occur due to its interaction with carbon dioxide. The sensor comprises PABA, an electrolyte, a gas-permeable membrane, electrodes, and other components (Figure 12). For the electrolyte, Nafion, a solid electrolyte, is used to prevent leakage and drying, issues that are common with liquid electrolytes. Nafion easily oxidizes, functioning as an acidic catalyst and an ion exchange resin. It aids PABA in detecting carbon dioxide by accelerating moisture absorption through its interaction with sulfonic acid, which PABA requires for moisture. When carbon dioxide passes through the gas-permeable membrane, it reacts with water in the electrolyte region to produce hydrogen carbonate and hydrogen ions (protons), which protonate PABA. As the partial pressure of carbon dioxide increases, so does the degree of protonation, leading to increased conductivity. This process results in a change in the pH of the electrolyte and, consequently, a change in potential. The advantages of this sensor include a simple physical dispersion process for electrode deposition, high environmental stability, and high water solubility during electrochemical polymerization. The sensor performs well at humidity levels between 20% and 70% and temperatures between 25 °C and 55 °C. It is capable of detecting carbon dioxide concentrations up to 2455 ppm [61].

#### 3.2.6. Citric Acid-Treated Chitosan-Based Carbon Dioxide Indicator

One of the challenges with general chitosan-based carbon dioxide indicators is the pH sensitivity of chitosan. If the pH sensitivity is too high, these indicators can react to environmental changes caused by factors other than carbon dioxide. To address this issue, a citric acid-treated chitosan-based carbon dioxide indicator has been developed. The indication method and detection mechanism of this new indicator are similar to conventional chitosan-based indicators, with the key difference being that the chitosan is treated with citric acid. Typically, chitosan has a dissolution critical point at pH 6 to 7, making it prone to dissolve even with minor environmental changes. However, when treated with citric acid, the nitrogen atoms in the amine group of chitosan bind to the carboxyl group of citric acid, resulting in a new dissolution critical point of pH 2.5 to 3.5. This modification allows for more effective detection of carbon dioxide generated inside food packaging [62].

#### 3.2.7. Sodium Caseinate, Pectin-Based Carbon Dioxide Indicator

**Figure 13:**
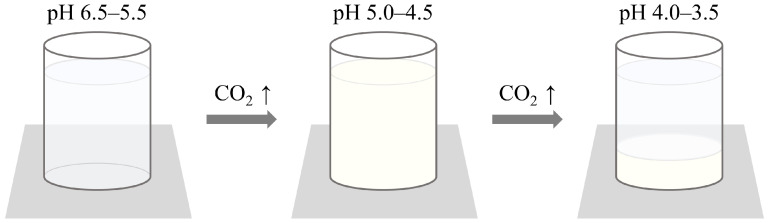
Detection and indication mechanism of the sodium caseinate and pectin-based carbon dioxide indicator. The indicator utilizes the pH sensitivity of sodium caseinate to detect carbon dioxide levels. At pH 6.5–5.5, the solution remains transparent. As carbon dioxide levels increase, causing the pH to drop to 5.0–4.5, the solution becomes opaque. Further increase in carbon dioxide results in a pH of 4.0–3.5, leading to precipitation. This three-stage indication mechanism allows for sensitive detection of carbon dioxide changes [55].

Sodium caseinate exhibits cohesiveness below its isoelectric point, similar to the characteristics of whey protein isolate (WPI) discussed in Section 3.2.4 [15,55]. An indicator made using sodium caseinate and pectin, a water-soluble polysaccharide, indicates the amount of carbon dioxide generated in three stages: transparent, opaque, and precipitated, depending on the pH (Figure 13). The detection mechanism relies on the change in solubility of sodium caseinate with pH changes. Pectin remains negatively charged on its surface regardless of pH, allowing control of transmittance by adjusting the isoelectric point of sodium caseinate through varying pectin concentrations. This indicator is advantageous as it is harmless to the human body and sensitively detects changes in food quality, making it easy for consumers to recognize these changes [55,56].

#### 3.2.8. Cresol Red Anion/Tetraalkylammonium Cation Ion Pair-Based Carbon Dioxide Indicator

The indicator is produced by mixing an ion pair material, formed through ion exchange between the alkali metal salt of cresol red and tetraalkylammonium halide, with an amine compound, polyethyleneimine, and a hydrophobic resin, then coating this mixture onto a substrate. The presence of carbon dioxide is indicated by a color change in the cresol red within the ion pair material [11,57]. The detection mechanism involves cation detection by the cresol red anion and a subsequent color change due to a shift in pH. The amine compound in the indicator creates pores and cellular structures within the film containing the hydrophobic polymer resin, allowing gaseous carbon dioxide to diffuse into the film. Furthermore, the indicator can detect carbon dioxide in the absence of moisture by identifying cations generated through the adsorption of gaseous carbon dioxide by the ion pair of cresol red anions. The main advantage of this indicator is its ability to detect carbon dioxide across different concentrations [57].

#### 3.2.9. Film Type Acidity Indicator

During kimchi storage, carbon dioxide is produced by fermentation due to microorganisms, and this carbon dioxide reacts with moisture to reduce pH. To provide consumers with information about this pH change, which is an important indicator of kimchi’s ripening, a film-type acidity indicator was developed. This indicator is composed of a carbon dioxide absorbent, an acidity regulator, a binding agent, and a dye. It is made in film form and visually indicates pH or acidity by the color change of the dye. The detection mechanism relies on measuring the pH change due to carbon dioxide. Calcium hydroxide, used as the carbon dioxide absorbent in the indicator, absorbs carbon dioxide and generates water. However, the strong basicity of calcium hydroxide makes it difficult to detect pH changes accurately. To address this, an acidity regulator was added to control the pH, thereby improving the accuracy of pH change detection. The film-type acidity indicator is primarily designed to measure changes in the acidity of kimchi. It is expected to be applicable to other food packaging as it can indirectly measure the generation of carbon dioxide by detecting pH changes due to carbon dioxide [11,58].

### 3.3. Ammonia Gas Indicator and Sensor

**Figure 14:**
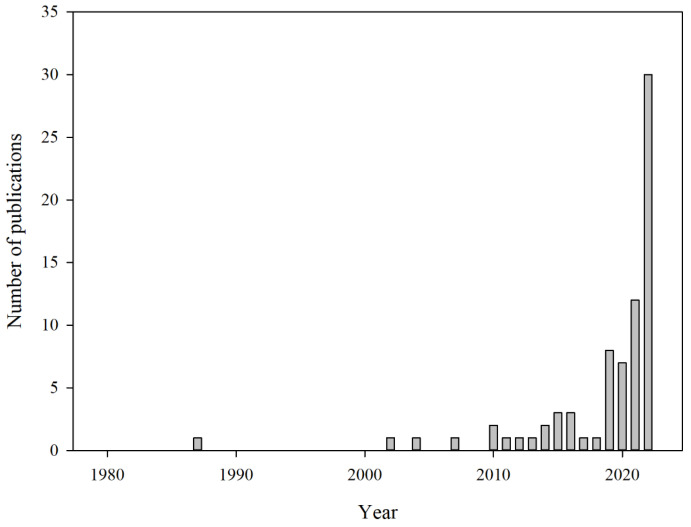
The annual number of publications related to ammonia indicators and sensors for food packaging has been indexed in PubMed up to 2022 [71]. A sharp rise in the number of publications has been observed after 2020, reflecting an increase in research interest in this specific area.

When high-protein foods like meat and fish deteriorate, volatile amines such as ammonia, dimethylamine, trimethylamine, and biogenic amines (BA) are produced, which are referred to as total volatile basic nitrogen (TVBN) [24,63,64]. The production of volatile amines results from the breakdown of amino acids by enzyme and microbial activity. These compounds contribute to the formation of off-flavors and serve as critical markers of food spoilage [17,24,65,66]. Given that ammonia is a key component of TVBN and a significant marker of spoilage, extensive research is being conducted to develop ammonia indicators and sensors for improved food quality control (Table 3, Figure 14) [24,63,67,68,69].

**Table 3:** Investigated ammonia gas indicators and sensors.

Indicator/ Sensor	Detection Method	Indication Method	Sensitivity		Detection Range or LOD		Reference
TiO_2_/Ti_3_C_2_T_x_ based sensor	Electron transfer	Electrical signal	1.26/ppm		156 ppt		[63]
Low density polyethylene-curcumin film	pH change	Color change	Ammonia concentration 0–80 µM	0.0388 nm/µM	0.18 µM (At 90% relative humidity)		[24]
			Ammonia concentration 100–240 µM	0.0124 nm/µM			
Silica-reinforced polydiacetylene (PDA) nanofiber mat	Internal conformational change	Color change Fluorescence intensity change	ECDA	41.68 counts/ppm	ECDA	6.95 ppm	[20]
Curcumin, curcumin analogs based colorimetric sensing label	Electron transfer	Color change Fluorescence intensity change	N/A		Bur-BF_2_	84.85 ppb	[70]
Fe (II) based colorimetric platform	Partial spin state change from HS to LS of Fe ion	Color change	N/A		105 ppb		[64]
Manganese oxide nanoparticle doped graphene-based ammonia sensor	pH change	Transparency change	N/A		N/A		
Rapid-response ionic-conducting laminate ammonia sensor	Electrode reaction	Electrical signal	N/A		N/A		
Cellulose nanocrystal-silver nanoparticle film-based indicator	Volatile material, silver nanoparticle chemical bonding	Color change	N/A		N/A		
Ruthenium-enhanced tungsten trioxide ammonia gas sensor	Ammonia dehydrogenation	Resistance value	N/A		N/A		

#### 3.3.1. TiO_2_/Ti_3_C_2_T_x_ Based Sensor

Current ammonia gas indicators and sensors have limitations in practical applications for real-time, in-situ monitoring due to low sensitivity, specificity, or inadequate automation levels. To address this issue, a film-type sensor was developed using a sensing material composed of a secondary transition metal compound, Ti_3_C_2_T_x_, and (001) TiO_2_, along with electrodes. Ti_3_C_2_T_x_, the most widely studied among MXenes (secondary transition metal compounds), has high specificity for ammonia at room temperature due to its large specific surface area, which allows it to absorb many termination groups. Additionally, Ti_3_C_2_T_x_ is suitable as a co-catalyst for photocatalysts because it accelerates electron and electron-hole transfer due to its excellent electrical conductivity and high carrier mobility. TiO_2_ is widely used as a gas detector and photocatalyst due to its eco-friendliness and excellent photocatalytic performance. These characteristics allow the sensor to lower its operating temperature under UV irradiation due to the generation of photogenerated electrons, which also improve sensitivity, reaction time, and recovery time. The sensor operates on the principle that ammonia gas reacts with oxygen ions to emit electrons, increasing the electron carrier concentration of the sensor components and thereby reducing the sensor’s resistance. After UV irradiation, the sensitivity of the indicator was measured at 1.26/ppm of ammonia gas, with a limit of detection of 156 ppt [63].

#### 3.3.2. Low-Density Polyethylene (LDPE)-Curcumin Film

**Figure 15:**
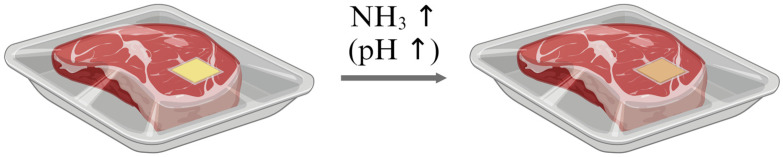
Detection mechanism of the LDPE-Curcumin film. As meat spoils, ammonia gas is released, causing a pH increase. This change in pH results in a color change in the curcumin dye within the film, indicating meat spoilage [24].

Among the various methods for fabricating indicators, incorporating natural dyes into hydrophilic polymeric films presents a challenge under conditions of high relative humidity, particularly during meat storage. High relative humidity can cause the dye within the film to elute and infiltrate the food. While the use of natural dyes (food colors) eliminates toxicity concerns, this infiltration compromises the effectiveness of the indicator. To address this issue, a low-density polyethylene (LDPE)-curcumin film was developed by embedding the natural pigment curcumin into a hydrophobic polymer film (LDPE). This indicator detects the presence of ammonia gas through a color change in the curcumin dye. The detection mechanism relies on the color change of the dye in response to pH changes caused by ammonia gas (Figure 15). This method is advantageous as it is non-toxic and cost-effective. The sensitivity of the indicator varies with ammonia gas concentration: for concentrations ranging from 0 to 80 µM, the sensitivity is 0.0388 nm/µM, and for concentrations between 100 and 240 µM, the sensitivity is 0.0124 nm/µM. Additionally, the LOD at 90% relative humidity was determined to be 0.18 µM [24].

#### 3.3.3. Silica-Reinforced Polydiacetylene (PDA) Nanofiber Mat

**Figure 16:**
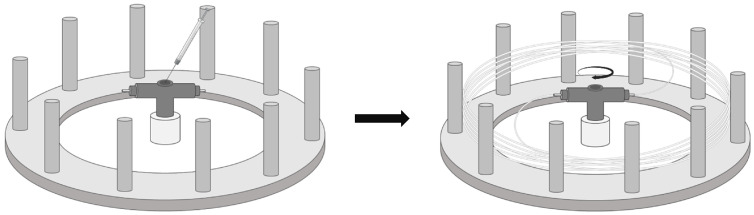
Schematic overview of Forcespinning^®^ technology for producing DA nanofibers. The precursor solution containing DA monomers is loaded into the reservoir (**left**), and the solution is extruded through a rapidly rotating spinneret to form nanofibers through centrifugal force (**right**) [20].

Meats and fish decay rapidly if not stored under optimal conditions. Due to health and economic concerns, there is increasing interest in the quality control of these foods. To facilitate real-time and in-situ monitoring, a silica-reinforced polydiacetylene (PDA) nanofiber mat was developed using PDA nanofibers through Forcespinning^®^ technology. This indicator detects the generation of ammonia gas by changes in color and fluorescence intensity. The detection mechanism involves an internal conformational change in PDA due to the electrostatic attraction between amine ions from the BA and carboxylate ions in PDA. When PDA nanofibers react with amine ions, they change color from blue to red and exhibit increased fluorescence. PDA comprises a hydrophobic hydrocarbon tail with a diacetylene (DA) triple bond and an amphiphilic polar head. The color of PDA changes when exposed to analytes or external stimuli such as pH, temperature, mechanical stress, or molecular bonds or when crosslinks are formed. Another characteristic of PDA is that during polymerization, the monomers tend to self-assemble, maintaining their structure. When the DA monomer is irradiated with UV rays at 254 nm, crosslinking occurs through a 1,4-addition reaction, forming an ene-yne cross-polymer chain. Indicators are fabricated using Forcespinning^®^ technology, which is gaining attention due to its ability to mass-produce fibers without high voltage and conductive spinning solutions (Figure 16). Forcespinning^®^ technology offers several advantages, including cost-effectiveness, time efficiency, easy processing, and a wide selection of non-conductive and conductive materials. The indicator clearly shows color changes and enables real-time, in-situ analysis. For the diacetylene monomer, both 10,12-pentacosadiynoic acid and 5,7-eicosadienoic acid (ECDA) were tested. The indicator made using ECDA showed better results for amine detection, with a sensitivity of 41.68 counts/ppm and a detection limit of 6.95 ppm [20].

#### 3.3.4. Curcumin, Curcumin Analogs Based Colorimetric Sensing Label

Natural dyes used in colorimetric indicators have good sensitivity to ammonia but are challenging to use due to their water-insoluble properties. A method to improve the dispersion of these dyes involves using an ethanol/water solution in a cellulose-based ink system. The indicator was produced through screen printing by combining curcumin, its analogs (Bur, Bur-BF_2_), and hydroxyethyl cellulose ink, which possesses excellent rheological properties. The indicator detects the presence of ammonia gas through changes in color and fluorescence intensity, with the detection mechanism based on electron transfer within the molecule. Curcumin, a polyphenol compound, is insoluble in water and exhibits bright yellow and green fluorescence under UV light. Due to these characteristics, it is widely used in pH detection devices. However, curcumin’s practicality is limited by its relatively short absorption/emission wavelength, low chemical stability, and poor photostability. Screen printing is an easy and cost-effective method to produce indicators that show food freshness through color or pattern changes. Achieving high-definition printing patterns requires good dispersion of components (thickener, dye, and solvent) in the ink system. Cellulose is used as a thickener, and among various types of cellulose, hydroxyethyl cellulose is preferred for its high biocompatibility, low toxicity, and excellent film-forming properties, which improve the rheological properties of the ink. The indicator not only changes color but also alters fluorescence intensity, with the Bur-BF_2_-based indicator demonstrating the highest sensitivity. The LOD was measured at 84.85 ppb [70].

#### 3.3.5. Fe (II) Based Colorimetric Platform

In recent years, the use of ammonia gas indicators for real-time and in-situ monitoring in food packaging applications has been constrained by challenges such as inadequate sensitivity, specificity, and measurement accuracy. To overcome these limitations, a non-porous colorimetric complex was developed. This indicator operates without energy at room temperature and has excellent reusability. The detection mechanism involves a partial change in the spin state of Fe (II) ions when exposed to gaseous amines. The surface of the Fe (II) complex undergoes a partial spin-state change from high-spin to low-spin upon adsorbing the analyte, which results in a color change of the indicator. The advantages of this indicator include energy-free operation at room temperature, high reusability, and the unique chemical and physical properties of the Fe (II) coordination complex, such as high catalytic activity, excellent specificity, sensitivity, and low cost. The LOD was measured to be 105 ppb [64].

#### 3.3.6. Manganese Oxide Nanoparticle Doped Graphene-Based Ammonia Sensor

Ammonia gas is generated not only from protein foods but also in fields such as livestock, agriculture, waste disposal sites, human excreta treatment plants, compound fertilizer manufacturing, and drug manufacturing processes. Since ammonia gas is toxic and harmful to the human body, real-time and in-situ monitoring of ammonia gas emissions is required. Therefore, an ammonia gas detection sensor incorporating a substrate, graphene sheet, and metal nanoparticles has been developed. This sensor indicates the presence of ammonia gas through changes in surface conductivity at room temperature. The detection mechanism involves changes in ion concentration within the electrolyte layer. Typically, graphene exhibits p-type conductivity, which causes electron transfer by adsorbing oxygen and water vapor on its surface. At room temperature, a layer of water molecules is adsorbed on the surface of a manganese oxide (Mn_3_O_4_) sensor, acting as a liquid electrolyte. When ammonia (NH_3_) is exposed to the surface of graphene doped with Mn_3_O_4_ nanoparticles, it penetrates the liquid electrolyte and reacts with water molecules, ionizing into NH_4_^+^ and OH^−^. The dissociation of NH_3_ molecules alters the ion concentration in the electrolyte layer by increasing ion conductivity and pH value. Consequently, when the graphene surface is exposed to ammonia gas, an electron emission reaction occurs, increasing the conductivity of the graphene sensor’s surface. The advantages of this sensor include its low cost and ease of manufacture. Additionally, it offers fast reaction and recovery times, excellent specificity for ammonia gas, reproducibility, and real-time detection capabilities [72].

#### 3.3.7. Rapid-Response Ionic-Conducting Laminate Ammonia Sensor

Recently, regulations regarding ammonia concentration have become more stringent, creating a need to enhance the responsiveness of ammonia sensors to comply with these new standards. Therefore, a measuring device that measures either the potential difference or the current between the plate-shaped sensor element, in which a pair of electrodes with different reactivity to ammonia is formed on the surface, and the electrode, as well as a sensor in which all the electrodes are exposed to the gas, were developed. The sensor indicates the presence of ammonia gas through an electrical signal, and the detection mechanism is based on the electrode reaction of ammonia. Ammonia gas passes through the solid electrolyte and moves from one side to the other side of the solid electrolyte of the sensor element. As a result, the electrode reaction of ammonia occurs, generating an electromotive force that correlates with the ammonia concentration between the electrodes. The electrode reaction for oxidizing ammonia is promoted at the first electrode, thereby generating an electromotive force between the pair of electrodes, with the first electrode acting as the anode and the second electrode as the cathode. Additionally, materials such as ZnO, SnO_2_, and In_2_O_3_ exhibit high oxidation activity against ammonia, which further promotes the electrode reaction for oxidizing ammonia. Consequently, a large electromotive force is generated between the electrodes, enabling more accurate detection of ammonia gas concentration based on the electromotive force [73].

#### 3.3.8. Cellulose Nanocrystal-Silver Nanoparticle Film-Based Indicator

Indicators should be attached to the inside of food packages to measure harmful volatile substances that can affect the quality of the food. To address this, a film-type indicator was developed, consisting of silver nanoparticles (AgNP) created by mixing and reacting cellulose nanocrystal and silver nitrate using natural materials. This indicator measures harmful volatile substances generated from food. The indicator indicates the degree of freshness through a color change in the film, which depends on the freshness of protein foods. The detection mechanism relies on the color change caused by alterations in the optical properties of the silver nanoparticles. When protein foods deteriorate, volatile substances such as hydrogen sulfide, methyl disulfide, and ammonia are produced. These substances form bonds with the silver nanoparticles, altering their optical properties and resulting in a color change. The advantage of this indicator is that it can be transparently applied to the inner surface of the packaging material, allowing it to be easily visible from the outside [74].

#### 3.3.9. Ruthenium-Enhanced Tungsten Trioxide Ammonia Gas Sensor

A gas sensor capable of detecting low concentrations of ammonia is necessary for quickly identifying and mitigating ammonia gas generated inside food packages. To meet this need, a gas sensor was developed using a sensing material prepared by adding a catalytic metal (ruthenium) to metal oxide (WO_3_). The resistance value of the sensor changes based on the ammonia concentration, with the detection mechanism being ammonia dehydrogenation. WO_3_ powder is produced by heat-treating W-sol, which is prepared by mixing tungstic acid (H_2_WO_4_) with hydrogen peroxide (H_2_O_2_) and stirring the mixture. Ru-sol is made by dissolving ruthenium acetylacetonate in ethanol. The WO_3_ powder and Ru-sol are mixed using an ultrasonicator, then the solution and powder are separated by centrifugation and dried to prepare the sensing material. When ammonia gas is adsorbed onto the WO_3_ particle surface, the electron carrier density increases, enhancing electrical conductivity and thus reducing the sensor’s resistance. The inclusion of catalytic metal ruthenium lowers the formation energy of ammonia gas on the WO_3_ surface, improving adsorption and desorption reactions, which in turn reduces the reaction and recovery times of the sensor. Notably, this sensor demonstrated high reactivity even at low concentrations of 5.0 ppm or less [75].

## 4. Conclusions

Gas indicators and sensors in smart packaging play a crucial role in informing consumers about potential food spoilage during storage and distribution. This review investigated various gas indicators and sensors, focusing on oxygen, carbon dioxide, and ammonia, and examined their detection method, indication method, sensitivity, and detection range or LOD. The findings suggest that gas indicators and sensors can provide easily interpretable information about harmful substances and food quality without opening the packaging. This capability is valuable for safety management and standardization policies in food packaging. However, applying these technologies to commercial food packaging presents several challenges. Current indicators and sensors need higher accuracy and precision, and improvements in material stability and safety are also required. Additionally, the investigated indicators lack measured characteristics such as sensitivity, detection range, and detection limits, making application difficult. These uncertainties limit their effectiveness for specific food types, necessitating further research to address these issues. High production costs also hinder mass production, necessitating the exploration of cost-effective materials and optimized manufacturing processes.

Despite these challenges, this review is expected to make a significant contribution to the application of these technologies in commercial food packaging by comprehensively providing the characteristics of indicators and sensors in the form of a table. The information presented in this review will not only help readers establish important criteria when selecting indicators and sensors suitable for specific food products but also provide valuable data that can be utilized in future research and development. These insights could ultimately lead to the development of more reliable and economically feasible gas indicators and sensors, thereby enhancing the safety and quality of food for both consumers and the food industry.

## Data Availability

No new data were created or analyzed in this study. Data sharing is not applicable to this article.
